# Sleep disruption is not observed with brain‐responsive neurostimulation for epilepsy

**DOI:** 10.1002/epi4.12382

**Published:** 2020-02-21

**Authors:** Leslie Ruoff, Beata Jarosiewicz, Rochelle Zak, Thomas K. Tcheng, Thomas C. Neylan, Vikram R. Rao

**Affiliations:** ^1^ San Francisco Veterans Affairs Health Care System San Francisco CA USA; ^2^ NeuroPace, Inc. Mountain View CA USA; ^3^ University of California San Francisco Sleep Disorders Center San Francisco CA USA; ^4^ Department of Psychiatry University of California San Francisco San Francisco CA USA; ^5^ Department of Neurology and Weill Institute for Neurosciences University of California San Francisco San Francisco CA USA

**Keywords:** arousal, brain‐responsive neurostimulation, epilepsy, polysomnography, RNS, sleep

## Abstract

**Objective:**

Neurostimulation devices that deliver electrical impulses to the nervous system are widely used to treat seizures in patients with medically refractory epilepsy, but the effects of these therapies on sleep are incompletely understood. Vagus nerve stimulation can contribute to obstructive sleep apnea, and thalamic deep brain stimulation can cause sleep disruption. A device for brain‐responsive neurostimulation (RNS^®^ System, NeuroPace, Inc) is well tolerated in clinical trials, but potential effects on sleep are unknown.

**Methods:**

Six adults with medically refractory focal epilepsy treated for at least six months with the RNS System underwent a single night of polysomnography (PSG). RNS System lead locations included mesial temporal and neocortical targets. Sleep stages and arousals were scored according to standard guidelines. Stimulations delivered by the RNS System in response to detections of epileptiform activity were identified by artifacts on scalp electroencephalography.

**Results:**

One subject was excluded for technical reasons related to unreliable identification of stimulation artifact on EEG during PSG. In the remaining five subjects, PSG showed fragmented sleep with frequent arousals. Arousal histograms aligned to stimulations revealed a significant peak in arousals just before stimulation. In one of these subjects, the arousal peak began before stimulation and extended ~1 seconds after stimulation. A peak in arousals occurring only after stimulation was not observed.

**Significance:**

In this small cohort of patients, brain‐responsive neurostimulation does not appear to disrupt sleep. If confirmed in larger studies, this could represent a potential clinical advantage of brain‐responsive neurostimulation over other neurostimulation modalities.

**Key Points**

- Polysomnography was performed in people with epilepsy treated with a brain‐responsive neurostimulation device
- Epileptiform brain activity preceded arousals and triggered stimulation by the device
- Arousals consistently preceded stimulations
- Brain‐responsive neurostimulation does not appear to disrupt sleep, a potential clinical advantage over other neurostimulation modalities

## 1 INTRODUCTION

Patients with drug‐resistant epilepsy^1^ who are not candidates for resective surgery^2^ may benefit from implanted neurostimulation devices—vagus nerve stimulation (VNS), thalamic deep brain stimulation (DBS), or brain‐responsive neurostimulation (RNS System)—adjunctive palliative treatments that can reduce seizures over time.^3^, ^4^ The advent of these three FDA‐approved devices has made neurostimulation for epilepsy a burgeoning field.^5^ With ever‐increasing clinical use of these devices, however, a key question concerns the possibility of unintended adverse effects of neurostimulation, especially on sleep.^6^ For example, VNS is associated with stridor and exacerbation of sleep‐disordered breathing, including obstructive sleep apnea (OSA),^7^, ^8^ possibly owing to effects on laryngeal motility.^9^ Thalamic DBS disrupts sleep in a dose (voltage)‐dependent manner,^10^ possibly helping to explain the adverse neuropsychiatric effects (memory loss, depression) associated with this therapy.^11^, ^12^, ^13^ Potential effects on sleep of RNS System treatment have been described only in a limited case report.^14^

The RNS System, comprising a cranially implanted programmable neurostimulator and two intracranial leads, delivers electrical stimulation in response to detection of abnormal (epileptiform) patterns of activity at the seizure focus/foci.^15^ Over time, median reduction in seizure frequency with brain‐responsive neurostimulation reaches 75%,^16^ and the therapeutic mechanism is thought to involve, in part, desynchronization of high‐frequency cortical rhythms.^17^ Whether this desynchronization affects sleep, which is dominated by slower, synchronized frequencies, remains unknown. Given the close, bidirectional relationship between sleep and epilepsy,^18^ and the fact that RNS System stimulations often peak during nocturnal hours when epileptiform activity is most frequent,^19^ it is essential for clinicians to know whether this therapy—intended to reduce seizures—might have counterproductive effects on sleep.

Here, we performed polysomnography (PSG) on six subjects who were implanted with the RNS System for treatment of medically refractory focal epilepsy. Stimulations delivered by the RNS System in response to detections of epileptiform activity were identified by artifacts on scalp electroencephalography (EEG).^20^ Arousal histograms aligned to stimulations were used to examine the temporal relationship between arousals and stimulations to test whether brain‐responsive neurostimulation disrupts sleep.

## 2 METHODS

### 2.1 Study participants

Study participants were selected from a cohort of 44 adults with medically refractory focal epilepsy who were implanted with the RNS^®^ System (Model RNS‐300M; NeuroPace Inc) for purely clinical indications. RNS System lead locations were determined by clinicians at time of implantation based on knowledge of the seizure onset zone(s). Inclusion criteria for study participation were as follows: age ≥ 18, implanted for >6 months, low (100‐500) or high (>2000) average daily stimulation rate (determined by review of the Patient Data Management System (PDMS), a secure, interactive, online repository for data collected by the neurostimulator^15^), and willingness and ability to tolerate overnight PSG for research purposes, the latter being the principal barrier to study recruitment. Exclusion criteria for the study were as follows: known central or obstructive sleep apnea (2 out of 44 RNS System patients had known OSA; both diagnosed prior to RNS System implantation), severe cognitive deficits or other barriers to providing informed consent, and stimulation being disabled on the device. The study protocol was approved by the Institutional Review Board at the University of California, San Francisco, and all subjects provided written informed consent.

### 2.2 Polysomnography

Study participants each underwent one night of ambulatory polysomnography (PSG) in a hotel setting using a 34‐channel portable recording device (Embla^®^ Titanium, Natus Medical Incorporated) and analyzed using Embla^®^ RemLogic™ sleep diagnostic software. Participants took their usual medications on the day of PSG. The standard American Academy of Sleep Medicine (AASM) recommended PSG montage was extended to include additional scalp electrodes placed according to the 10‐20 International System and used to assist with identifying stimulation artifact. PSG recordings included EEG (Fp1, Fp2, F3, F4, F7, F8, C3, C4, T3, T4, T5, T6, M1, M2, O1, O2, Fz, and a common reference (Cz) allowing for the flexibility to view EEG in additional montages), left and right electrooculogram (EOG), submental chin and bilateral leg electromyogram (EMG), EKG, chest and abdominal respiratory inductance plethysmography (RIP) belts, flow sensors (nasal pressure transducer and oronasal thermistor), position sensor, and pulse oximetry. A full PSG was conducted to have the ability to associate arousals with respiratory or limb movements as well as stimulation artifact. Participants did not use continuous positive airway pressure therapy at any time during PSG.

Sleep stages and arousals were manually scored in 30‐second epochs by the same registered polysomnographic technologist (author LR) and further reviewed epoch by epoch with a Board‐certified sleep physician (author RZ) in accordance with AASM Guidelines v2.5.^21^ An *arousal* was defined as an abrupt shift of EEG frequencies, including theta, alpha, and/or frequencies greater than 16 Hz (but not spindles), that lasted at least 3 seconds, with at least 10 seconds of stable sleep preceding the change. The timestamps of electrical stimulations delivered by the RNS System in response to epileptiform activity were also manually scored and identified by artifacts on scalp EEG,^20^ and studies were scored blind to RNS System ECoG. Timestamps of stimulations saved by the RNS System neurostimulator were not used as the primary data source because (a) they had a lower temporal resolution (0.5 seconds), and (b) they were incomplete for some subjects because of data loss due to storage limitations on the neurostimulator. For those subjects for whom a full night of RNS‐saved stimulation timestamps was available (S4 and S6), the RNS‐saved timestamps were used to verify that all stimulations were accounted for and that no spurious ones were added in the manually scored EEG artifact‐identified list of stimulation times. Due to circadian and multiday variation in RNS stimulation rate,^22^ the number of RNS stimulations manually scored in the overnight PSG could exceed the daily average stimulation rate.

RNS System detection and stimulation parameters were not changed from clinical settings (Table S1) prior to PSG. Due to limited memory capacity of the neurostimulator, stored data were downloaded several hours before and several hours after PSG to minimize risk of data loss by overwriting. Memory constraints also underlie the fact that, although the neurostimulator continuously senses brain activity, it cannot continuously store ECoG; rather, storage of short (typically 90‐second) four‐channel ECoG is triggered at prespecified times of day and/or by occurrence of events likely to indicate seizures, such as prolonged detections of epileptiform activity. ECoG records stored during the night of the study were later aligned with corresponding portions of the PSG based on clock time and stimulation artifacts.

### 2.3 Data analysis

To create peristimulation arousal histograms for each subject, we first defined two windows of time (±60 seconds and ±4 seconds) centered on each manually identified stimulation timestamp. Stimulations occurring within one window of the beginning or end of the PSG recording were excluded from analysis. The 120‐seconds and 8‐seconds windows were divided into 1‐seconds and 100‐ms bins, respectively. Using the timestamps (to the nearest millisecond) of each stimulation, any arousals that occurred within 60 seconds (or 4 seconds) of that stimulation incremented the arousal count in the time bin corresponding to that arousal's timestamp relative to that stimulation. This was repeated for each stimulation, producing a histogram of arousal counts in each time bin surrounding each stimulation (ie, producing a peristimulation arousal histogram). The average rate of arousals (per stimulation, per minute) in the prestimulation vs poststimulation half of each time window was computed for each patient.

### 2.4 Statistical analyses

To test whether arousal counts on either side of time‐aligned stimulations were significantly greater than expected by chance given the baseline rates of stimulations and arousals, we used a nonparametric shuffle test. The order of inter‐arousal intervals for each subject was randomly permuted 100 000 times using the *randperm* function in MATLAB (MathWorks). Each time, we used the randomly permuted inter‐arousal intervals to create a new time series of arousal times that had the same mean rate and the same distribution of inter‐arousal intervals as the original series of arousal times, but for whom any relationship with the stimulation times existed only by chance. We then recomputed the peristimulation arousal histogram for each shuffle iteration, obtaining a new “shuffled” arousal rate for each window before and after the time‐aligned stimulations. Together, these shuffled‐arousal rates provide a null distribution of the rates of peristimulation arousals expected by chance for each window. To obtain an estimated *P*‐value for the actual arousal rate in each window (ie, the probability of having obtained the actual arousal rate by chance), we computed the percentage of null (shuffled‐arousal) rates that equaled or exceeded the actual arousal rate.

## 3 RESULTS

### 3.1 Subject characteristics

From a cohort of 44 patients at UCSF implanted with the RNS System for treatment of medically refractory focal epilepsy, we identified 6 subjects (4 females; age range 21‐50; Table 1) who met inclusion/exclusion criteria and consented to study participation. Subjects had been implanted with the RNS System for at least 6 months (to avoid a period of time postimplantation when intracranial recordings are unstable^23^), did not have a known sleep disorder, and were willing to undergo one night of PSG. Epilepsy etiologies were diverse, mean duration of epilepsy was 19.3 ± 11 years, and all subjects were being treated with two or three AEDs (Table 1). Regarding other medications with potential effects on sleep, one subject (Subject 4) used alprazolam as needed for treatment of panic disorder, and another subject (Subject 5) was taking a stimulant (methylphenidate) for treatment of attention deficit disorder.

**Table 1:** Subject characteristics

Subject #	Age	Gender	Epilepsy onset age	Epilepsy etiology	AEDs	Other relevant meds	SOZ	RNS lead 1 location	RNS lead 2 location	Lead laterality	Mesial temporal (M), Neocortical (N)	RNS Stims/d
1	50	M	16	Cryptogenic	PHT, CLB	‐	Bilateral hippocampi	L hippocampus	R hippocampus	B	M	2741
2	37	F	14	Eclampsia	OXC, LEV, CZP	‐	Bilateral hippocampi	L hippocampus	R hippocampus	B	M	4574
3	21	M	15	FCD	LCM, CZP	‐	Lateral temporal	Heschl's gyrus	Posterior superior temporal gyrus	L	N	2247^a^
4	35	F	9	Cryptogenic	LEV, CBZ	alprazolam	Lateral frontal	Mesial frontal	Dorsolateral frontal	R	N	309
5	32	F	12	MCD	CLB, LTG, ZNS	methylphenidate	Lateral occipital	Dorsolateral occipital	Ventrolateral occipital	R	N	158
6	25	F	18	PVNH	TPM, CBZ	‐	Posterior temporal	Periventricular nodule + overlying cortex	Hippocampus	L	M, N	493

Note

‘RNS Stims/d’ indicates mean number of daily therapies delivered by the RNS System over 3 months prior to night of PSG.

Abbreviations: B, bilateral; CBZ, carbamazepine; CLB, clobazam; CZP, clonazepam; F, female; FCD, focal cortical dysplasia; L, left; LCM, lacosamide; LEV, levetiracetam; LTG, lamotrigine; M, male; MCD, malformation of cortical development; OXC, oxcarbazepine; PHT, phenytoin; PVNH, periventricular nodular heterotopia; R, right; SOZ, seizure onset zone; TPM, topiramate; ZNS, zonisamide.

a PSG for this subject could not be scored and used for analysis because, for unclear reasons, RNS stimulation artifact was not reliably detectable in scalp recordings.

### 3.2 RNS System characteristics

RNS System intracranial lead placement is configured based on the location and extent of the seizure onset zone(s).^24^ In our subjects, RNS System leads were mesial temporal (N = 2, bilateral in both), neocortical (N = 3), and both (N = 1) (Table 1). Detection and stimulation parameters programmed on the neurostimulator are customized for each individual and iteratively tuned by clinicians based on electrographic data and patient‐reported seizure frequency (Table S1). Stimulation involves delivery of constant‐current, charge‐balanced, square‐wave pulses, typically in short (eg, 100 ms), high‐frequency (eg, 200 Hz) trains. Because stimulation is delivered only in response to detections of epileptiform activity, the number of daily stimulations varies widely across patients, ranging from tens to thousands of stimulations per day (typically amounting to several minutes of total daily stimulation).^25^, ^26^ Given the possibility that sleep effects might depend on the total amount of stimulation, we included subjects with both low (100‐500) and high (>2000) average daily stimulation rates (Table 1).

### 3.3 Polysomnography

Subjects underwent one night of hotel‐based PSG. RNS System stimulation artifact on scalp EEG^20^ enabled identification of stimulation times, and sleep stages and arousals were scored in accordance with AASM guidelines.^21^, ^27^ Subject 1 met criteria for new diagnosis of OSA based on apnea‐hypopnea index (AHI) of 45/h, and Subject 2 had an elevated Respiratory Disturbance Index (RDI) but did not meet polysomnographic criteria for OSA (RDI = 12.9/h with a normal AHI of 3.2/h) (Table 2). Not unexpectedly, these two subjects also had the most frequent RNS stimulations per hour of sleep (324.4/h and 213.6/h, respectively). The arousal indices associated with RNS stimulation (RNS stimulation arousal indices) were low at 1/h or fewer and were lower than the overall arousal indices and the rate of arousals associated with respiratory events or PLMS. Subject 3 was excluded from subsequent data analysis for technical reasons (stimulation artifact could not be identified on EEG during PSG, so peristimulation arousal histograms could not be created; see Table 1).

**Table 2:** Polysomnography results

	Subject					
	1	2	3	4	5	6
Sleep architecture						
TST (minutes)	411	595.5	248.0	522.5	543.0	579.5
SPT (minutes)	523	683.3	402.3	562.2	616.8	585.3
SE (%)	77.4	86.0	52.4	88.2	86.6	98.3
SM (%)	78.6	87.2	61.7	92.9	88.0	99.0
SOL (minutes)	7.7	9.5	70.8	30.3	10.0	4.3
REM sleep latency (minutes)	259	130.0	280.5	120.5	50.5	56.0
WASO (minutes)	112.3	87.8	153.8	39.7	73.8	5.8
N1 (% TST)	22.9	12.9	17.1	6.1	5.2	5.9
N2 (% TST)	38.6	57.9	29.2	59.3	68.0	39.8
N3 (% TST)	24.6	8.8	28.4	8.0	0.0	20.0
REM (% TST)	14.0	20.4	25.2	26.5	26.8	34.3
Sleep fragmentation						
Awakenings (count)	113	79	40	26	30	10
Awakenings (index)	16.5	8.0	9.7	3.0	3.31	1.04
Total Arousal (count)	146	265	56.0	164	317	231
Arousal index (events/h)	21.31	26.7	13.6	18.8	35.0	25.4
AHI	45	3.2	3.6	4.1	2.2	N/A^a^
RDI	45.1	12.9	4.4	4.1	2.2	2.4
LM index (events/h)	0.0	4.3	9.5	13	11.8	5.3
LM arousal index (event/h)	0.0	0.7	1.2	2.9	1.0	3.0
PLMS index (events/h)	0.0	0	2.7	0.9	0.7	0.4
PLMS arousal index (events/h)	0.0	0.0	0.0	0.0	0.0	0.0
Visually identified stimulation artifact						
RNS stimulation (count)	2828	2432	81	34	82	352
RNS stimulation (TST index)	412.8	245.0	19.6	3.9	9.0	38.7
RNS stimulation (SPT index)	324.4	213.6	12.1	3.6	8.0	36.1
RNS stimulation arousal index	1.0	0.2	1.0	0.0	0.3	0.2

Abbreviations: AHI, apnea–hypopnea index; LM, limb movement; PLMS, periodic limb movements of sleep; RDI, respiratory disturbance index; SE, sleep efficiency; SM, sleep maintenance = TST/(time from sleep onset to sleep offset); SOL, sleep onset latency; SPT, sleep period time; TST, total sleep time; WASO, wake after sleep onset.

a Subject refused overnight pulse oximetry so only an RDI was used.

### 3.4 Distribution of arousals relative to RNS System stimulations

**Figure 1:**
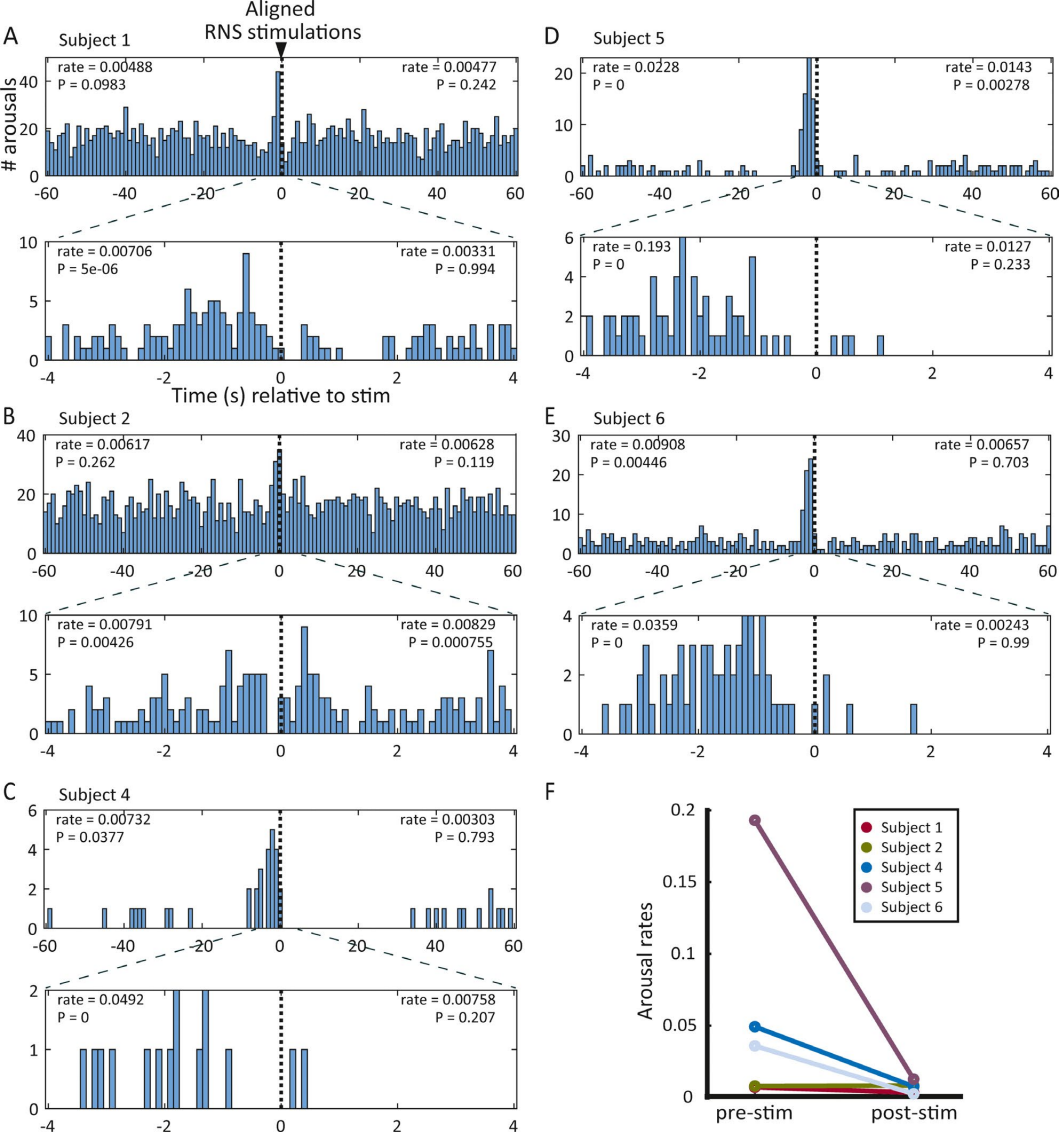
Temporal relationship between arousals and RNS System stimulations for each subject. A–E, Histograms showing frequency distribution of arousals relative to stimulations aligned at *t* = 0 for subjects 1 (A), 2, (B), 4 (C), 5 (D), and 6 (E). In each panel, plots show 60‐s (top) and 4‐s (bottom) windows before and after aligned stimulations. Axis labels in A also apply to B–E. All subjects demonstrated a peak in arousals just before stimulation except Subject 2 (B), who showed an increase in arousals both before and after RNS stimulation. Arousal rates shown for each pre‐ and poststimulation window are in units of arousals/RNS stimulation/minute. The *P*‐values shown in each pre‐ and poststimulation window indicate the probability of observing the actual arousal rate (or higher) by chance given the baseline stimulation and arousal rates for that subject, obtained by randomly shuffling the actual inter‐arousal intervals 100 000 times. F, Plot summarizing the prestimulation (left) and poststimulation (right) arousal rates in the 4‐s windows shown in A‐E. Each bar connects the prestimulation to the poststimulation arousal rate data for one patient. Note that the prestimulation rates tend to be higher than the poststimulation arousal rates (n.s.), which is the opposite of the expected effect if stimulation consistently caused arousals

We reasoned that if brain‐responsive neurostimulation causes sleep disruption, stimulations should consistently precede arousals. For example, in a study of the effects of thalamic DBS on sleep, Voges and colleagues found that arousals were more frequent following the onset of stimulation than during nonstimulation periods.^10^ Unlike thalamic DBS, an open‐loop device that delivers scheduled intermittent stimulation (eg, regular cycles of 1‐minutes ON, 5‐minutes OFF), the RNS System is a closed‐loop device that delivers stimulation irregularly—and often with brief, isolated bursts—in response to detection of abnormal patterns of brain activity. Thus, we compared the frequency of arousals before vs after individual stimulations by plotting the distribution of arousals time‐aligned to all stimulations within each subject (Figure 1).

In all five subjects, the distribution of arousals showed a significant peak just *before* the onset of stimulation (Figure 1A–E, *P*‐values for significance shown in left side of plots). In one subject (Subject 2), who also happened to have the highest rate of daily stimulations (Table 1), the arousal peak began before stimulation and extended to ~1 second after stimulation (Figure 1B). Across subjects, the rate of arousals before stimulations tended to be higher than the rate of arousals after stimulations (Figure 1F). Together, these results are inconsistent with what would be expected if RNS stimulations caused arousals (see *Models to explain observed results* below).

### 3.5 Correlating ECoG and polysomnography

**Figure 2:**
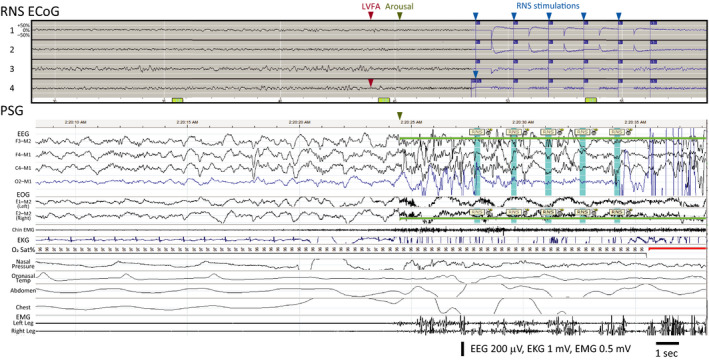
Epileptiform activity can precede arousal and RNS System stimulation. Data from Subject 4 showing electrocorticogram stored by the RNS System (‘RNS ECoG’) aligned with corresponding polysomnogram (‘PSG’). ECoG Channels 1 and 2 show bipolar recordings from a mesial frontal strip lead, and Channels 3 and 4 show bipolar recordings from a dorsolateral frontal strip lead. PSG shows the subject in stage N3 with an arousal (green highlight) at the end of the epoch and five visually identified stimulation artifacts (blue highlight) labeled “RNS.” The respiratory and leg channels show the absence of any significant respiratory or movement events preceding the arousal. This subject's typical ictal pattern involves attenuation and emergence of low‐voltage fast activity (‘LVFA’) in Channel 4 (red arrowhead), which, here, is followed by arousal from sleep (green arrowhead) and then a sequence of five stimulations (maximum allowed by neurostimulator; blue arrowheads) triggered by detection of this epileptiform pattern (indicated by blue portion of ECoG trace). The temporal sequence of events suggests that epileptiform activity caused both arousal and stimulation

**Figure 3:**
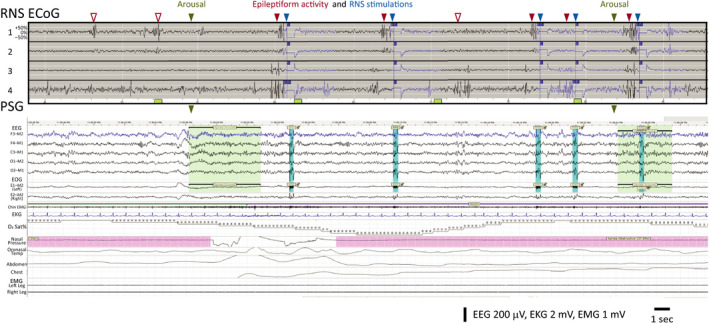
RNS System stimulations can precede and follow arousals. Data from Subject 1, aligned RNS ECoG and PSG as in Figure 1. ECoG Channels 1 and 2 are bipolar recordings from the left hippocampus, and Channels 3 and 4 are recorded from the right hippocampus. ECoG shows frequent bilateral independent epileptiform discharges, some that are undetected by the RNS System (empty red arrowheads) and some that are detected (filled red arrowheads), triggering stimulation (blue arrowheads; note stimulation artifact on top two scalp EEG channels in PSG). PSG captures two arousals (green arrowheads), and stimulations are observed shortly after the first arousal and shortly before the second arousal. The temporal sequence of events suggests that at least some arousals in this subject are independent of stimulations

Our study design afforded a rare opportunity to correlate sleep features on PSG with concurrent intracranial recordings.^14^, ^28^, ^29^ To examine the relationships among stimulations, arousals, and epileptiform activity in more detail, we visually compared ECoGs recorded by the RNS System (subject 1: two 180‐seconds ECoGs; all other subjects: two 90‐seconds ECoGs) with their temporally corresponding portions of PSG. This was particularly informative in one subject (Subject 4) with frontal lobe epilepsy who, at baseline, has multiple electrographic seizures every night characterized by low‐voltage fast activity (LVFA). Inspection of stored ECoGs from this subject revealed that stimulations triggered by detection of LVFA occur after the arousal (Figure 2). Given the limited number of ECoGs stored during PSG for each subject, it is difficult to generalize this sequence of events, but this example nevertheless illustrates one way in which arousals can precede stimulations (Figure 1). Similarly, inspection of ECoG from another subject with bitemporal epilepsy and frequent interictal epileptiform discharges (Subject 1) revealed that only a subset of discharges are detected by the RNS System, and that stimulations are delivered before and after arousals (Figure 3). This example illustrates one way in which arousals can peak before and after stimulations (Figure 1B). None of the stored ECoGs revealed evidence of stimulation causing arousals on corresponding PSG.

### 3.6 Models to explain observed results

**Figure 4:**
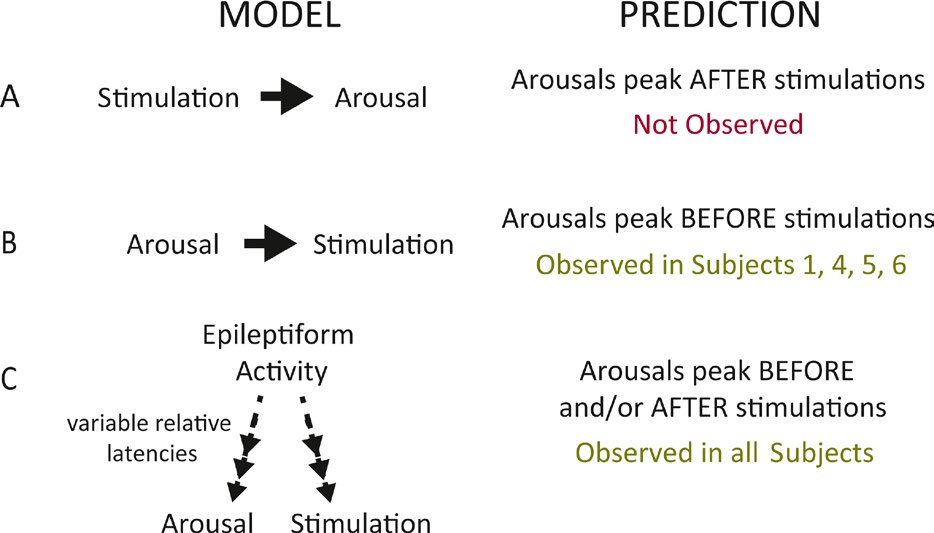
Models to explain observed results. A, If stimulation causes arousals, then more arousals should follow stimulations than expected by chance, and there should be a higher rate of arousals after than before RNS stimulations. We did not observe either of these outcomes in any patient. B, If arousals trigger brain‐responsive neurostimulation, then arousals should peak before stimulations. This is consistent with observations in all subjects, but it does not entirely explain Subject 2's results, whose arousal peak starts before and extends after stimulation. C, A model in which epileptiform activity causes both arousals and RNS stimulations with variable latencies is consistent with the pre‐ and peristimulation arousal peaks observed in all subjects, including Subject 2

The relative temporal distributions of epileptiform activity, stimulations, and arousals do not prove any causal relationships, but they do provide correlative information that can inform the relative likelihood of different hypothetical causal interdependencies among these variables (Figure 4). The hypothesis we set out to test was whether brain‐responsive neurostimulation causes arousals (Figure 4A). Under this scenario, we would have expected to observe more arousals following stimulations than expected by chance and a higher rate of arousals after rather than before RNS stimulations. We did not observe either of these outcomes in any patient (Figure 1).

Instead, we observed the opposite trend (arousals tending to peak *before* stimulations), which led us to formulate two other possible (not mutually exclusive) models. In one model (Figure 4B), arousals might trigger brain‐responsive neurostimulation if, for example, the RNS System is programmed in such a way that it incidentally detects brain activity associated with arousals in addition to the intended detection of epileptiform activity. This model predicts that arousals should peak before stimulations, consistent with observations in all subjects, but it does not entirely explain Subject 2's results, whose arousal peak starts before and extends after stimulation. In another possible model (Figure 4C), epileptiform activity causes both arousals^30^, ^31^, ^32^ and RNS stimulations^33^ with variable latencies. This model is consistent with both the pre‐ and peristimulation arousal peaks observed in all subjects, including Subject 2 (whose detection to stimulation latency may have been longer than the other subjects, causing some of the arousals to occur just after stimulation).

## 4 DISCUSSION

Here, by obtaining PSG in a small cohort of subjects implanted with the RNS System, we provide evidence that brain‐responsive neurostimulation of neocortical and mesial temporal targets does not disrupt sleep. Across subjects with diverse epilepsies and wide‐ranging rates of stimulation, we found that the distribution of arousals typically peaks just before, rather than after, stimulation. These results are inconsistent with what would be expected if RNS stimulations caused arousals. Instead, these pre‐ and peristimulation arousal peaks are consistent with the well‐known phenomenon that epileptiform activity itself is often followed by arousals,^30^, ^31^, ^32^ and the fact that epileptiform activity detected by the RNS System^33^ triggers stimulation at variable latencies across and within subjects.

Taken together with our comparison of intracranial ECoG activity with its corresponding PSG, our results favor a mixture of two models: one in which epileptiform activity causes both arousals and stimulations, and one in which arousals themselves may trigger brain‐responsive neurostimulation. We cannot rule out the possibility that some of the poststimulation arousals observed in Subject 2 may have been caused by the stimulations, and that the prestimulation arousals in this subject were explained by some mixture of the other two models. However, we find this possibility less likely given the more parsimonious explanation, consistent with all patients' results, that epileptiform activity causes both arousals and stimulations with variable latencies. Thus, in this small cohort of patients, stimulation with the RNS System does not appear to cause arousals. If confirmed in larger studies, this could represent a potential clinical advantage of brain‐responsive neurostimulation over other neurostimulation modalities.

RNS System stimulations typically peak during nocturnal hours,^19^ which may relate to sleep stage‐dependent facilitation of epileptiform discharges,^34^ to aberrant sleep rhythms at the seizure focus/foci,^35^, ^36^ and/or to circadian rhythms in interictal activity and seizures.^22^, ^37^ Yet, there is scant existing literature on the relationship between brain‐responsive neurostimulation and sleep. Our findings are consistent with a prior case report that did not find obvious effects of RNS System stimulation on progression through sleep stages,^14^ but this study involved a single subject with only mesial temporal leads, did not examine arousals in relation to stimulations, and did not correlate ECoG with PSG data.

Much of what is known about neurostimulation and sleep derives from the use of DBS to treat movement disorders like Parkinson's disease.^38^ DBS of some brainstem structures, including the subthalamic nucleus and the pedunculopontine nucleus, is generally associated with improvement in sleep quality,^39^, ^40^ but development of a sleep disorder is possible too,^41^ and stimulation of other deep targets can worsen sleep.^42^ The therapeutic mechanisms of VNS and DBS are thought to involve brainstem pathways^5^, ^43^ that also play important roles in sleep, but RNS System leads are typically placed in the neocortex or hippocampus, where the effects of stimulation on sleep are less clear. Although we found no evidence that RNS System stimulations disrupt sleep, we cannot determine whether this relates to anatomically favorable lead locations or, hypothetically, to favorable aspects of the subjects' stimulation parameters (Table S1). Furthermore, given that PSG was abnormal in most subjects (Table 2) and baseline (ie, before RNS System implantation) PSG was not available, we cannot rule out the possibility that RNS stimulation has local chronic effects that cause sleep instability and reduce sleep quality,^44^ even though individual stimulations do not appear to disrupt sleep acutely (on the timescale of seconds–minutes).

Our study has several other limitations. First, the sample size was relatively small. Although our center has one of the largest cohorts of patients implanted with the RNS System in the country, recruitment for this study was limited by strict inclusion and exclusion criteria and by prospective subjects' willingness to undergo overnight testing. Still, we were encouraged by the consistency of findings across subjects with diverse epilepsy etiologies, lead locations, and stimulation rates. Second, the fact that stimulations are directly triggered by detection of epileptiform activity, which itself can cause arousals,^30^, ^31^ complicates interpretation of the relationship between these variables. Ideally, for experimental purposes, stimulation would be applied randomly during PSG to test whether stimulation causes arousals independent of epileptiform activity. However, this functionality is not possible with the neurostimulator, which is designed to stimulate only in response to specific, typically epileptiform, ECoG events. Third, sleep under laboratory testing conditions can be atypical,^45^ though the comfortable environment of hotel‐based PSG may mitigate this concern in our study. Fourth, multidien (multiday) variation in the rate of interictal epileptiform activity^22^ (and thus in the rate of RNS stimulations) may limit generalizability of one‐night studies, though the consistent relationship between stimulations and arousals despite wide variation in mean stimulation rates across subjects (Table 1) helps to mitigate this concern. Fifth, whereas VNS and DBS involve lead placement in consistent locations (left vagus nerve and bilateral anterior thalamic nuclei, respectively), RNS System lead placements and stimulation pathways (ie, anode/cathode designations of the eight intracranial electrodes and the neurostimulator canister) are highly heterogeneous,^24^ and, across subjects, epileptogenic networks likely have variable relationships to the anatomy and physiology of sleep. Although our subjects represented various common lead placements (mesial temporal and/or neocortical, unilateral, or bilateral), we cannot exclude the possibility that some other lead placements (eg, thalamic) might disrupt sleep.^46^, ^47^ Finally, it is likely that not every stimulation delivered by the RNS System was identifiable on the surface EEG, and manually scored timestamps were not able to be verified for those subjects for whom a full night of RNS System‐saved stimulation times was not available (S1, S2, and S5). We expect that any missed stimulations are not biased relative to the timing of arousals, except when arousals are accompanied by myogenic and movement artifacts, which might mask artifacts caused by stimulations. Thus, in theory, we may have missed more RNS System stimulations *after* arousals than before arousals, which would lead to *underestimation* of our central observation, that is, arousals tending to peak before stimulations.

Our findings should motivate larger studies on the effects of neurostimulation on sleep.

PSG in patients with thalamic RNS System leads could unravel whether the divergent effects on sleep of thalamic DBS and hippocampal/neocortical brain‐responsive neurostimulation relate to distinct stimulation targets, stimulation timing (open‐loop versus closed‐loop), or other factors. A greater mechanistic understanding of sleep physiology and the effects of stimulation on brain networks will inform development of next‐generation devices with improved side effect profiles.

## CONFLICT OF INTEREST

BJ and TKT are employees of, and hold stock options at, NeuroPace, Inc. VRR is a paid consultant for NeuroPace, Inc, but does not have equity or ownership in the company. Other authors declare no relevant conflicts of interest. We confirm that we have read the Journal's position on issues involved in ethical publication and affirm that this report is consistent with those guidelines.
