# Supplementary material for: Sleep disruption is not observed with brain‐responsive neurostimulation for epilepsy
Source: Epilepsia Open. 2020 Feb 21;5(2):155–65. doi: 10.1002/epi4.12382 (PMC7278540; doi:10.1002/epi4.12382)
Supplement: Supplementary file 1 [file EPI4-5-155-s001.docx]

**Stimulation Waveform Parameters**

| **Subject #** | **RNS Lead 1 Location** | **RNS Lead 2 Location** | **Stimulation Pathway Tx1-5**  **(Lead 1)(Lead 2)(canister)** | **Current Intensity**  **(mA)** | **Frequency (Hz)** | **Pulse Width (µs)** | **Burst Duration**  **(ms)** | **Charge Density**  **(µC/cm^2^)** |
| --- | --- | --- | --- | --- | --- | --- | --- | --- |
| 1 | L hippocampus | R hippocampus | B1: ( )(0000)(+)  B2: (0000)( )(+) | 4.5 | 200 | 120 | 100 | 1.7 |
| 2 | L hippocampus | R hippocampus | B1: (+-+-)(0000)(0)  B2: (0000)(+-+-)(0) | 2.5 | 200 | 160 | 100 | 2.5 |
| 3 | Heschl's gyrus | Posterior superior  temporal gyrus | B1: ( )(++++)(0)  B2: (++++)( )(0) | 2.0 | 200 | 160 | 100 | 1.0 |
| 4 | Mesial frontal | Dorsolateral frontal | B1: ( )(++++)(0)  B2: (++++)( )(0) | 1.5 | 200 | 160 | 200 | 0.8 |
| 5 | Dorsolateral occipital | Ventrolateral occipital | B1: ( )(++++)(0)  B2: (++++)( )(0) | 5 | 200 | 160 | 200 | 2.5 |
| 6 | Periventricular nodule  + overlying cortex | Hippocampus | B1: (++--)(0000)(0)  B2: (--++)(+-+-)(0) | B1: 2.0  B2: 3.0 | 200 | 160 | 100 | B1: 2.0  B2: 1.5 |

Stimulation parameters programmed on neurostimulator during night of PSG. ‘Stimulation pathway Tx1–5’ indicates anode / cathode designations of the four electrodes contacts on Lead 1 and Lead 2, and the neurostimulator canister for each of a maximum of ﬁve sequential Therapies delivered by the neurostimulator in response to detection of abnormal patterns of activity. Each Therapy comprises two bursts of current, Burst 1 (B1) and Burst 2 (B2), that can be independently conﬁgured. Stimulation waveform parameters deﬁne charge-balanced, biphasic, square-wave pulses and are identical for B1 and B2, except where indicated. Charge density is calculated by dividing charge delivered per phase by electrode surface area.
